# Tumor Drug Distribution after Local Drug Delivery by Hyperthermia, In Vivo

**DOI:** 10.3390/cancers11101512

**Published:** 2019-10-09

**Authors:** Helena C. Besse, Angelique D. Barten-van Rijbroek, Kim M.G. van der Wurff-Jacobs, Clemens Bos, Chrit T.W. Moonen, Roel Deckers

**Affiliations:** Center of Imaging Sciences, University Medical Center Utrecht, Heidelberglaan 100, 3584 CX Utrecht, The Netherlands; angelique.barten@hu.nl (A.D.B.-v.R.); c.bos@umcutrecht.nl (C.B.); R.Deckers-2@umcutrecht.nl (R.D.)

**Keywords:** tumor drug distribution, spatial drug distribution, temperature sensitive liposomes, hyperthermia, chemotherapy, non-temperature sensitive liposomes

## Abstract

Tumor drug distribution and concentration are important factors for effective tumor treatment. A promising method to enhance the distribution and the concentration of the drug in the tumor is to encapsulate the drug in a temperature sensitive liposome. The aim of this study was to investigate the tumor drug distribution after treatment with various injected doses of different liposomal formulations of doxorubicin, ThermoDox (temperature sensitive liposomes) and DOXIL (non-temperature sensitive liposomes), and free doxorubicin at macroscopic and microscopic levels. Only ThermoDox treatment was combined with hyperthermia. Experiments were performed in mice bearing a human fibrosarcoma. At low and intermediate doses, the largest growth delay was obtained with ThermoDox, and at the largest dose, the largest growth delay was obtained with DOXIL. On histology, tumor areas with increased doxorubicin concentration correlated with decreased cell proliferation, and substantial variations in doxorubicin heterogeneity were observed. ThermoDox treatment resulted in higher tissue drug levels than DOXIL and free doxorubicin for the same dose. A relation with the distance to the vasculature was shown, but vessel perfusion was not always sufficient to determine doxorubicin delivery. Our results indicate that tumor drug distribution is an important factor for effective tumor treatment and that its dependence on delivery formulation merits further systemic investigation.

## 1. Introduction

Chemotherapy is one of the main treatment modalities of cancer. A commonly used chemotherapeutic agent is doxorubicin, which is used for the treatment of soft tissue sarcoma, among others [1,2,3,4]. As such, doxorubicin contributes to successful tumor treatment, but it also causes serious toxicities in non-tumor tissue, especially in the heart [5,6,7]. These toxicities are a dose limiting factor for doxorubicin and could lead to suboptimal treatment of the tumor and to compromised efficacy [8].

A common approach to reduce chemotherapy related toxicities is to encapsulate the drug in nanomedicine [9], such as liposomes [10,11]. Liposomes preferentially accumulate in the tumor tissue driven by the enhanced permeability and retention (EPR) effect [12]. After accumulation of the liposomes in the tumor, the chemotherapeutic agent diffuses from these liposomes over the course of days [13].

In 1995, DOXIL was the first Food and Drug Administration (FDA) approved doxorubicin containing liposome [14]. Preclinical experiments had demonstrated that DOXIL treatment reduced doxorubicin accumulation in the heart [15] and increased survival compared to free doxorubicin (DOX) treatment [16]. In humans with metastatic breast cancer, though, DOXIL treatment did decrease toxicity, but survival remained comparable to that of DOX treatment [11]. The observation that DOXIL treatment failed to increase survival in these patients has been attributed to inter- and intra-patient variations of the tumor EPR effect [17] and to the slow diffusion of doxorubicin from the liposomes, resulting in limited bioavailability of the drug [18].

The bioavailability of the drug in the tumor can be improved by combining temperature sensitive liposomes (TSL) with local hyperthermia, as first proposed in 1978 by Yatvin et al. [19]. The quick release of the encapsulated drug from TSLs is caused by increased permeability of the liposomal membrane for temperatures exceeding a threshold, its phase transition temperature [20]. Preclinical treatment with TSLs containing doxorubicin in combination with hyperthermia improved efficacy [21,22,23] and increased doxorubicin concentration gradients in the tumor [21,24,25,26]. 

A TSL containing doxorubicin available for clinical studies is ThermoDox [27]. ThermoDox releases 60% of the encapsulated doxorubicin within 20 seconds at a temperature of 41.3 °C, its phase transition temperature [28]. Currently, ThermoDox is in a phase III clinical trial for hepatocellular carcinoma in combination with tumor radiofrequency (RF) ablation (clinicaltrials.org identifier: NCT02161562). Previous clinical trials have already shown the safety and the feasibility of ThermoDox treatment in combination with RF ablation and hyperthermia [29,30,31,32]. 

Not only absolute drug concentration in the tumor, but also the spatial drug distribution of the drug is an important factor to achieve effective tumor treatment [33]. For example, Torok et al. showed that a more homogeneous spatial distribution of anti-angiogenic receptor tyrosine kinase inhibitors correlated with improved antitumor and anti-vascular effects [34]. Cesca et al. demonstrated that larger anti-tumor activity of paclitaxel after bevacizumab is due not necessarily to overall tumor drug levels but to better drug penetration and more homogenous drug distribution [35] A promising approach to improve the drug distribution and the drug concentration in the tumor is by TSL in combination with hyperthermia. TSL in combination with hyperthermia results in a more homogeneous spatial doxorubicin concentration [25], increased doxorubicin concentrations at larger distances from the vessels [24], and increased doxorubicin concentrations in the whole tumor [21,25,26] compared to DOX. Although a couple of landmark papers provided evidence for heterogeneous drug distribution after ThermoDox treatment, these studies only investigated the doxorubicin distribution in a qualitative way [25] or only in a small region of the tumor [24].

Here, the drug distribution after treatment with different doses of clinically available TSL containing doxorubicin, ThermoDox, was compared with clinically available non-temperature sensitive liposomes containing doxorubicin (NTSL), DOXIL, and free doxorubicin, DOX. Only ThermoDox treatment was combined with hyperthermia, since ThermoDox has to be combined with hyperthermia for triggered drug release, whereas DOX and DOXIL are, in daily clinical practice, always applied in the absence of hyperthermia. To investigate the efficacy of the different formulations, the tumor growth was monitored after treatment. Since drug distribution has an effect on the tumor growth, the drug distribution was studied quantitatively in whole tumor sections. After treatment with the different formulations, the drug distribution was investigated in terms of spatial doxorubicin distribution and doxorubicin concentration in the tumor and by doxorubicin concentration in relation to the perfused tumor vessels. Finally, the microscopic effect of the doxorubicin distribution in the tumor on tumor cell proliferation was investigated.

## 2. Results

### 2.1. Tumor Growth and Survival

**Figure 1:**
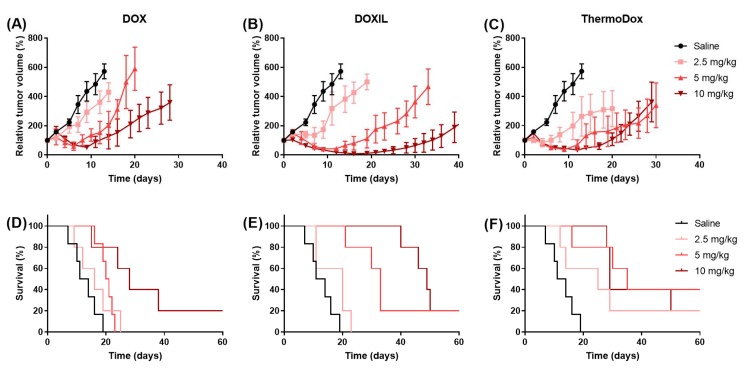
Tumor growth and survival (“tumor volume five times larger than the initial tumor volume”). Relative tumor volume and survival of tumors treated with saline, doxorubicin (DOX) (**A**,**D**), DOXIL (**B**,**E**), and ThermoDox (**C**,**F**) at a dose of 2.5, 5, and 10 mg/kg.

Figure 1A–C shows tumor growth of mice in the different experimental groups. Tumor growth was decreased with respect to controls in animals receiving DOX, DOXIL, and ThermoDox treatments. A dose dependent decrease in tumor growth was observed after DOX, DOXIL, and ThermoDox treatment, although after treatment with ThermoDox at doses of 5 and 10 mg/kg, the tumor growth was comparable. At 2.5 and 5 mg/kg, the largest growth delay was obtained after ThermoDox treatment, whereas at 10 mg/kg, the largest growth delay was obtained after DOXIL treatment.

Figure 1D–F shows the survival of mice (i.e., time to reach tumor volume five times larger than the initial tumor volume) in the different experimental groups. Survival was related to the tumor growth; in all cases, the mouse was sacrificed because of reaching the humane endpoint for tumor growth. Survival was increased with respect to the controls in animals receiving DOX, DOXIL, and ThermoDox treatments, which was significant at doses of 5 and 10 mg/kg. A dose dependent increase in survival was observed after DOX, DOXIL, and ThermoDox treatments with the exception that ThermoDox treatment at doses of 5 and 10 mg/kg, which resulted in comparable survival and comparable result for tumor growth. A significant dose dependent increase in survival (*p* < 0.05) was observed after treatment with DOX and DOXIL between doses of 2.5 and 10 mg/kg and 5 and 10 mg/kg. For ThermoDox treatment, no significant differences in survival were observed between the different doses. At a dose of 5 mg/kg, survival was significantly increased (*p* < 0.05) after DOXIL and ThermoDox treatments compared to DOX treatment. No significant difference in survival was observed between DOXIL and ThermoDox treatments at any dose.

Toxicities were only observed after ThermoDox treatment. One mouse died immediately after injection of 10 mg/kg ThermoDox and was excluded from the study. Treatment with 5 and 10 mg/kg ThermoDox resulted in a maximum weight loss of 7% relative to the weight immediately before treatment in three and five mice, respectively. The weight loss was classified as mild toxicity; hence, these mice were not withdrawn from the study. Since no weight loss was observed after ThermoDox treatment with 2.5 mg/kg in combination with hyperthermia, the mild toxicity observed after ThermoDox treatment with 5 and 10 mg/kg in combination with hyperthermia was most likely not related to the hyperthermia treatment but caused by the high local doxorubicin concentrations in the tumor bearing leg. 

### 2.2. Qualitative Distributions of Doxorubicin, Perfusion, Hypoxia, and Dividing Cells

**Figure 2:**
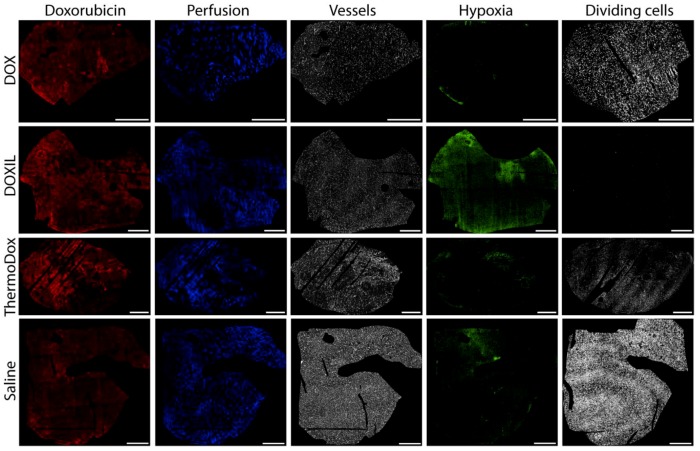
Different stainings of tumors treated with 5 mg/kg DOX, DOXIL, and ThermoDox. Tumors stained for doxorubicin, perfusion, all vessels, hypoxia, and proliferating cells. The scale bar is 1 mm.

Figure 2 and Figures S1–S3 show the distribution of doxorubicin, perfusion, vessels, hypoxia, and dividing cells in tumors after DOX, DOXIL, and ThermoDox treatment at different injected doses and saline (control). After all treatments, areas with high doxorubicin intensities, i.e., doxorubicin containing areas, were heterogeneously distributed over the whole tumor with spatially varied doxorubicin intensities. The percentage of doxorubicin containing area varied as a function of the injected dose and the treatment formulation; it was highest for the largest injected dose and for tumors treated with ThermoDox and DOXIL.

In all tumors, the vessels were homogeneously distributed throughout the tumor slice; however, only parts of these vessels were perfused. These perfused areas were heterogeneously distributed in the tumor slice. The doxorubicin containing areas were mainly located in areas that were perfused. However, after treatment with all formulations, there were also tumor areas that were perfused but did not contain doxorubicin. The hypoxic areas were heterogeneously distributed throughout the tumor slice. In addition, the percentage of hypoxic areas in the tumors differed between the tumors. The hypoxic areas were correlated with areas that did not contain doxorubicin. The dividing cells were also heterogeneously distributed throughout the tumor slice. Areas with few dividing cells were strongly correlated with doxorubicin containing areas.

### 2.3. Qualitative Doxorubicin Distribution

**Figure 3:**
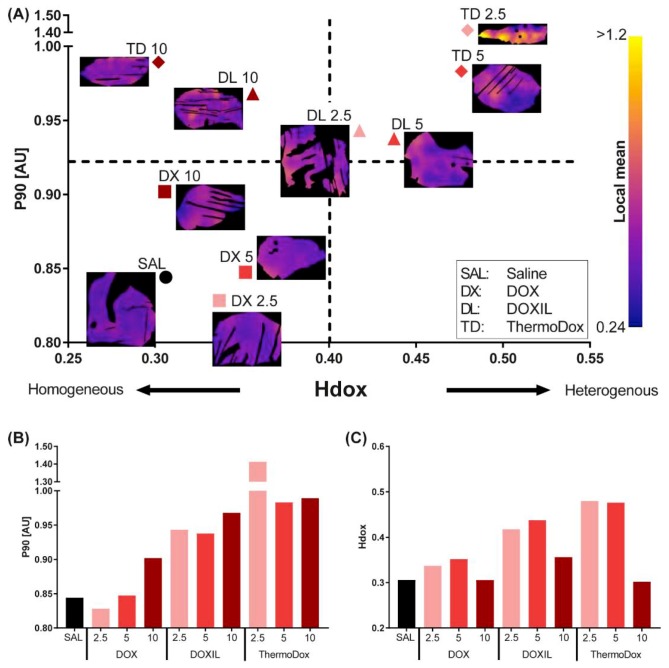
The 90th percentile of the doxorubicin concentration (P90) and the doxorubicin heterogeneity parameter (Hdox) over the whole tumor. Tumors treated with saline (SAL), DOX (DX), DOXIL (DL), and ThermoDox (TD) at 2.5, 5, and 10 mg/kg. The P90 (**A**,**B**) and the Hdox (**A**,**C**) were calculated over the whole tumor.

Figure 3A presents the correlation between the 90th percentile (P90) of the doxorubicin concentration and the doxorubicin heterogeneity parameter (Hdox) over the whole tumor slice in a scatter plot. High and low P90 values represent high and low tumor doxorubicin concentrations, respectively; high and low Hdox values represent heterogeneous and homogeneous spatial doxorubicin distribution, respectively. In the control tumor, both the P90 and the Hdox were low compared to DOX, DOXIL, and ThermoDox treated tumors, as expected.

The Hdox and the P90 were arbitrarily divided in high and low values at 0.4 and 0.92, respectively (Figure 3A). The untreated tumor (saline) and the tumors treated with DOX at all doses showed a low doxorubicin concentration that was homogeneously distributed in the tumor. Tumors treated with DOXIL and ThermoDox at doses of 2.5 and 5 mg/kg showed a high doxorubicin concentration that was heterogeneously distributed in the tumor. Tumors treated with DOXIL and ThermoDox at a dose of 10 mg/kg finally showed a high doxorubicin concentration that was homogeneously distributed throughout the tumor.

Overall, a trend was observed that P90 increased with increasing injected dose, although the tumor treated with 2.5 mg/kg ThermoDox was surprisingly high (Figure 3A,B). For all formulations, the spatial doxorubicin distribution was more homogeneous after treatment with 10 mg/kg than for doses of 2.5 and 5 mg/kg (Figure 3A,C). Doses of 2.5 and 5 mg/kg resulted in similar spatial doxorubicin distributions.

The P90 of ThermoDox treated tumors was increased compared to DOX and DOXIL treated tumors at all injected doses. The spatial doxorubicin distribution was more heterogeneous after ThermoDox treatment compared to DOX and DOXIL treatments at doses of 2.5 and 5 mg/kg. At a dose of 10 mg/kg, the spatial doxorubicin distribution after ThermoDox treatment was comparable with DOX treatment and more homogeneous compared to DOXIL treatment.

### 2.4. Doxorubicin Distribution Relative to Vessels

**Figure 4:**
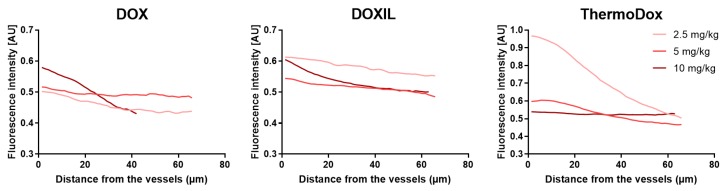
Doxorubicin intensities relative to the vessels over the whole tumor. The doxorubicin intensity relative to the vessels was determined after DOX (**A**), DOXIL (**B**), and ThermoDox (**C**) treatment at injected doses of 2.5, 5, and 10 mg/kg.

Figure 4 shows the doxorubicin intensity in relation to the distance of all perfused vessels in the whole tumor. In all tumors, the doxorubicin intensity close to the vessel was in the range of 0.5 and 0.6 AU except for the tumor treated with ThermoDox at a dose of 2.5 mg/kg, which was surprisingly high. In all tumors, the doxorubicin intensity declined with increased distance from the perfused vessels. However, no clear correlation was observed between the injected dose and the doxorubicin intensity in relation to the perfused vessels.

Since DOX, DOXIL, and ThermoDox treatments resulted in spatially varied doxorubicin intensities in the tumor, Figure 2 and Figures S1–S3, tumor locations with high and low doxorubicin concentrations were independently analyzed for doxorubicin intensity in relation to the perfused vessels (Figure S4). Doxorubicin intensity in relation to the perfused vessels in locations with high doxorubicin concentrations and in the whole tumor showed a similar trend, i.e., the doxorubicin intensity decreased with an increase in distance from the perfused vessels, and no clear correlation was observed between injected dose and doxorubicin intensity in relation to the perfused vessels. However, the absolute doxorubicin intensities were higher in locations with high doxorubicin concentrations compared to the whole tumor at all investigated distances from the perfused vessels. In locations with low doxorubicin concentrations, the doxorubicin intensity hardly changed with increased distance from the perfused vessel. In addition, the absolute doxorubicin intensities in areas with low doxorubicin concentrations were lower compared to the absolute doxorubicin intensities in the whole tumor at all investigated distances from the perfused vessels, as expected.

### 2.5. Cell Proliferation

**Figure 5:**
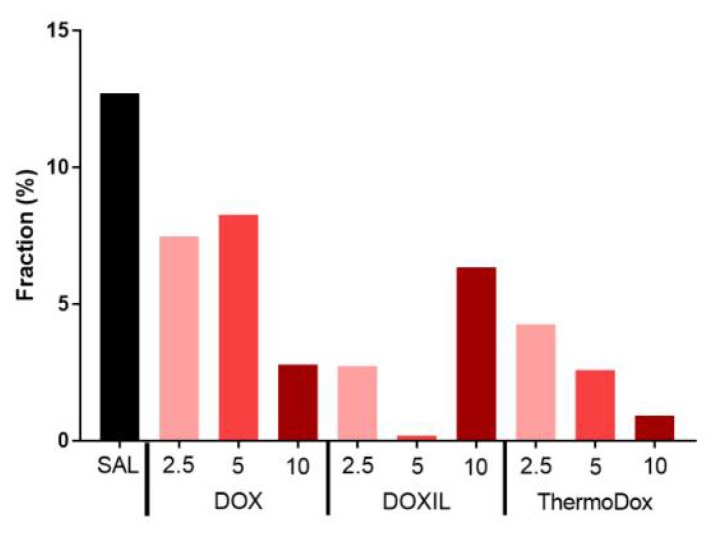
Dividing cell fraction over the whole tumor. The dividing cell fraction was calculated after tumors treated with SAL, DOX, DOXIL, and ThermoDox at doses of 2.5, 5, and 10 mg/kg. The tumor treated with DOXIL at a dose of 10 mg/kg was unexpectedly high.

Figure 5 shows the dividing cell fraction in tumors from the different experimental groups. The dividing cell fraction was reduced with respect to the control tumor after treatment with DOX, DOXIL, and ThermoDox. Overall, the dividing cell fraction decreased with increased injected dose for all formulations, although the dividing cell fraction in the tumor treated with 10 mg/kg DOXIL was unexpectedly high. The largest decrease in cell proliferation was obtained by DOXIL followed by ThermoDox and DOX at all doses.

**Figure 6:**
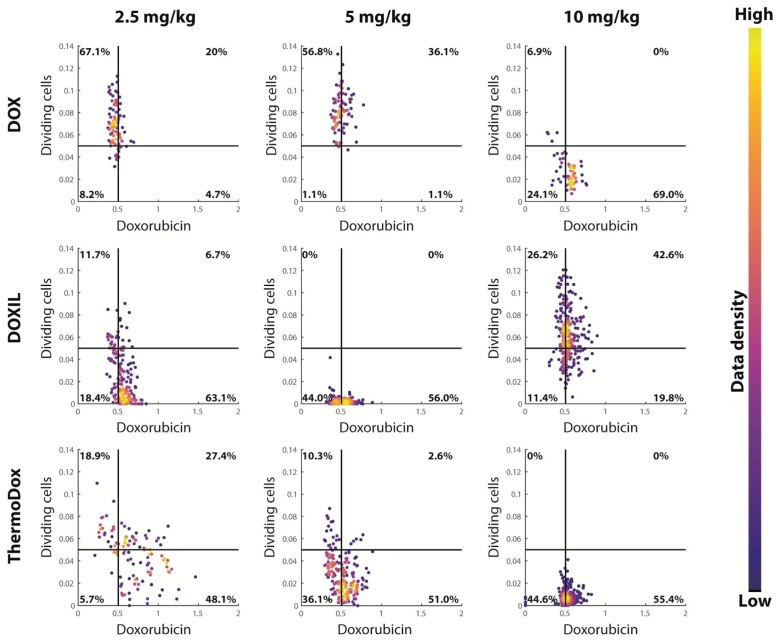
Correlation between dividing cell fraction and doxorubicin intensity. For each region in the tumor, the dividing cell fraction and the mean doxorubicin intensity were determined and are represented by one dot in the scatterplot. The color of the dots represents the data density of the locations. Dividing cell fraction below 0.05 and doxorubicin intensity below 0.5 were considered low.

Visual inspection of Figure 2 and Figures S1–S3 shows that areas with high doxorubicin intensity were correlated with reduced cell proliferation. This correlation was further quantitatively analyzed. Figure 6 presents, for each tumor, the dividing cell fraction and the doxorubicin intensity evaluated at each 200 × 200 pixel square of the tumor image, presented by a dot in the scatter plot. Generally, for each tumor, the dots were clustered. Overall, the dividing cell fraction per square decreased with increasing dose, as was already observed for whole tumor slices (Figure 5). In addition, an increasing trend was present between doxorubicin intensity and injected dose similar to that observed in the whole tumor slices (Figure 3B). Overall, the dots were mainly located either at high doxorubicin intensity and low dividing cells or at low doxorubicin intensity and high dividing cells.

### 2.6. Tumor Microenvironment

**Figure 7:**
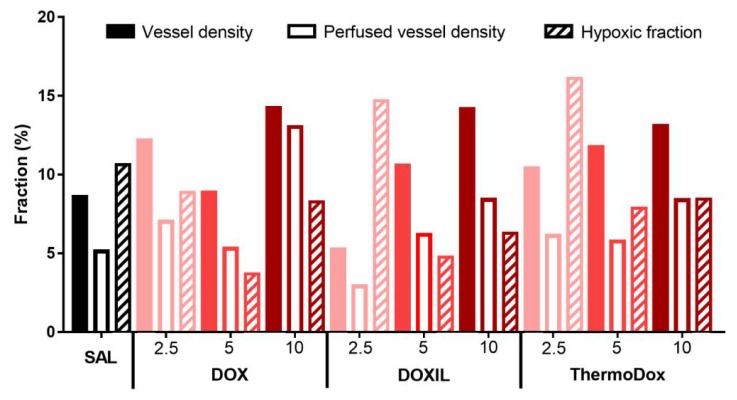
Vessel density, perfused vessel density, and hypoxic fraction. For each tumor, the vessel density (closed bars), the perfused vessel density (open bars), and the hypoxic fraction (striped bars) were determined in the whole tumor section.

The doxorubicin distribution in the tumor not only depends on the doxorubicin formulation and the injected dose, it also depends on the tumor microenvironment [33]. The microenvironments of the tumors were compared by vessel density, perfused vessel density, and the hypoxic fraction (Figure 7). The perfused vessels were a subset of all vessels, and 50–85% of all vessels were perfused. The vessel fraction, the perfused vessel fraction, and the hypoxic fraction differed between all tumors. In all tumors, the vessel density, the perfused vessel density, and the hypoxic fraction were between 5–15%, 3–14%, and 4–14%, respectively. No correlation was observed between vessel fraction, perfused vessel fraction, or hypoxic fraction and injected formulation or dose.

## 3. Discussion

Several studies have shown that tumor drug distribution is an important factor for effective tumor treatment [33,34,35]. TSL combined with hyperthermia is a promising approach to increase the drug distribution and the drug concentration in the tumor [24,25,36,37]. Therefore, we studied in more detail the efficacy and the drug distribution in the tumor after treatment with different doxorubicin formulations, namely, ThermoDox (clinically available TSL with triggered release using hyperthermia), DOXIL (clinically available NTSL), and DOX, with different injected doses. The largest growth delay was obtained after ThermoDox treatment at lowest and intermediate doses, whereas at the highest dose, the largest growth delay was obtained after DOXIL treatment. We observed that an increase in injected dose resulted in decreased tumor growth and a more homogeneous spatial doxorubicin distribution for each formulation. In addition, an increase in injected dose of DOX and DOXIL treatment resulted in increased doxorubicin concentrations in the tumor.

A dose dependent decrease in tumor growth was observed after ThermoDox, DOXIL, and DOX treatment, as shown before [38]. ThermoDox treatment with doses of 5 and 10 mg/kg resulted in comparable tumor growth, most likely since the efficacy of ThermoDox did not further increase at doses larger than 5 mg/kg. The formulation that resulted in the largest growth delay depended on the injected dose. At doses of 2.5 and 5 mg/kg, the largest reduction in tumor growth was present after ThermoDox treatment. This is in line with previous studies demonstrating that TSLs such as ThermoDox result in greater anti-tumor effects compared to NTSLs such as DOXIL [21,39]. At the highest investigated dose, i.e., 10 mg/kg, DOXIL treatment resulted in the largest growth delay in this study. We have no specific explanation for these differences; most likely, they are related to the maximum doxorubicin concentration and the area under the curve of the bioavailable doxorubicin concentration in the tumor.

As qualitatively observed, ThermoDox, DOXIL, and DOX treatments resulted in a heterogeneous doxorubicin distribution with spatially varied doxorubicin concentrations. This is in line with other studies that show a heterogeneous drug distribution in the tumor after treatment with DOX [40] and other drugs [41,42]. These doxorubicin containing areas were mainly present in close proximity to perfused vessels. Interestingly, there were also areas with perfused vessels that showed low doxorubicin intensities. We cannot explain why perfused vessels, in spite of similar appearance, showed very different surrounding drug distributions, yet comparable observations have been described in literature for DOX [43] and other drugs [42,44].

Many methods have been proposed to quantify the tumor drug distribution [45,46,47]. However, these methods are based on either drug concentration or spatial drug distribution. In our opinion, both drug concentration and spatial drug distribution are important to characterize the doxorubicin distribution in the tumor. Therefore, we separated our analysis in tumor concentration and spatial drug distribution and devised measures to quantify these from a fluorescence image.

A dose dependent increase in doxorubicin concentration in the tumor was present after DOX and DOXIL treatments, as observed before in whole tumors [48]. However, doxorubicin concentrations in the tumor after ThermoDox treatment were inconsistent in this study. For all formulations, an increase in injected dose resulted in a more homogeneous spatial doxorubicin distribution, as was qualitatively observed before for other drugs [44,49]. 

For all injected doses, the doxorubicin concentration in the tumor after ThermoDox treatment was increased compared to DOX treatment. Differences in spatial drug distribution between DOX and ThermoDox treatments were dose dependent. At doses of 2.5 and 5 mg/kg, the spatial doxorubicin distribution after treatment with ThermoDox was heterogeneous compared to DOX treatment, while at a dose of 10 mg/kg, the spatial doxorubicin distributions of ThermoDox and DOX were comparable. These results were not in line with Ranjan et al. [25], in which the spatial doxorubicin distribution at a dose of 5 mg/kg after ThermoDox treatment was homogeneous compared to DOX treatment. However, Ranjan et al. [25] qualitatively described the doxorubicin distribution and did not distinguish between the doxorubicin concentration and the spatial doxorubicin distribution in the tumor.

Although an increased injected dose corresponded well with decreased tumor growth, more homogeneous doxorubicin distribution, as well as increased tumor doxorubicin concentration, the relation between tumor growth and tumor doxorubicin concentration and distribution was less distinct. For each formulation, the group with the slowest tumor growth corresponded with the group with the highest tumor doxorubicin concentration and the most homogenous spatial drug distribution, as expected, since heterogeneous tumor drug distribution has been shown to decrease tumor treatment efficacy [33,34,50]. Interestingly, we did observe a correlation between areas with high doxorubicin concentrations and reduced dividing cells in all groups. Apparently, this first drug response was inconclusive to determine the final efficacy of the different formulations. It is known that the doxorubicin concentration in the tumor varies over time, and that these variations in concentrations are formulation dependent [18]. Therefore, further research should be conducted on variations in doxorubicin distribution in the tumor over time with single and repeated dosing and on the correlation between these time dependent variations and the efficacy of the different formulations.

In this study, no correlation was observed between the doxorubicin intensity in relation to the distance from the vessels and the injected dose. This was unexpected, since it was shown by Rhoden et al. [51] that an increase in injected dose increased the drug concentration both in close proximity to the vessels and at larger distances from the vessels 24 hours after administration of the antibodies. However, these experiments were performed with antibodies, and therefore our results cannot be directly compared with the results of Rhoden et al.

Drug distribution in the tumor depends on the properties of the drug formulation but is also influenced by the tumor microenvironment [52]. No differences were observed between vessel density, perfused vessel density, and hypoxic fraction between the tumors in the different formulations and injected doses. Therefore, we concluded that the observed differences in drug distribution between the different formulations and the injected doses were not related to the differences in tumor microenvironment. In addition, note that the hypoxic fraction was relatively low [53,54,55,56] and the vessel density and the perfused vessel density were rather high [57,58] in the investigated tumors. Hence, in this study, a well perfused tumor model was used.

A couple of limitations in this study were that, in the drug delivery part of this study, only one slice per tumor per treatment was chosen for further analysis; therefore, doxorubicin distribution was only investigated in a small part of one tumor per treatment and not in three-dimensional space. Consequently, a few important factors such as the out of plane vessels and the drug distribution in the whole tumor were not taken into account. In addition, since one tumor per treatment was analyzed, a bias could have occurred. In this study, 24 hours after treatment, animals were euthanized and immunohistochemistry was performed, since cell proliferation was included as an early marker for tumor response. However, it is known that doxorubicin concentration is preferably assessed between 30 and 60 minutes after administration of DOX [59] and between 24 and 48 hours after administration of DOXIL [15,40,59].

Doxorubicin fluorescence intensities were used as a measure of doxorubicin concentrations in the tumor. However, the doxorubicin fluorescence intensity not only depends on the doxorubicin concentration, it also depends on the interaction of the doxorubicin with molecules in its environment, such as the histones and the DNA [60,61]. We assumed that, in all tumors, the doxorubicin molecules were similarly affected by the molecules in their environment.

In this study, hyperthermia was used as a trigger to release doxorubicin from ThermoDox; however, hyperthermia can also have direct effects on the tumor, such as enhancing blood flow and permeability of the vasculature [62]. These effects may also lead to increased drug concentrations [62,63] and altered drug distribution [64,65,66] in the tumor. However, in this study, we used hyperthermia only in combination with ThermoDox, since hyperthermia is required for drug release by ThermoDox. In addition, in the clinic, DOX and DOXIL are mainly applied without hyperthermia.

## 4. Materials and Methods 

### 4.1. Materials

DOX (Accord Healthcare, Utrecht, The Netherlands), DOXIL (Janssen-Cilag BV, Breda, The Netherlands), and ThermoDox (Celsion Corporation, Lawrenceville, NJ, USA) were obtained at a doxorubicin concentration of 2 mg/mL.

### 4.2. Cell Culture

HT1080 (human fibrosarcoma) cells (obtained from ATCC, ATCC CCL-121, Rockville, MD, USA) were cultured in Minimum Essential Medium (MEM, Gibco, Grand Island, NY, USA) supplemented with 292 mg/L L-glutamine (Sigma, St. Louis, MO, USA), 110 mg/L sodium pyruvate (Sigma), and 10% fetal bovine serum (Sigma F7524). Cells were cultured at 37 °C in 5% CO2 in an air humidified incubator and regularly tested for mycoplasma contamination.

### 4.3. Animal Model

Seven to nine weeks Balb/c immune deficient mice (CAnN.Cg-Foxn1nu/Crl, Charles River) were housed in a 12 hour light/dark cycle. Food and water were available ad libitum. Tumors were inoculated by subcutaneous injection of 1 × 10^6^ cells [phosphate buffered aline (PBS):matrigel (354230, Corning, Corning, NY, USA), volume 1:1] in the hind leg. All animal experiments were approved by the Utrecht Animal Experimental Committee (DEC Utrecht, project number: 2013.III.09.066, approval date: 17 October 2013).

### 4.4. Chemotherapy Treatment

Mice with tumor volumes between 50 and 100 mm^3^ were randomized and received a single intravenous (i.v.) injection into the tail vein with different doxorubicin formulations (i.e., DOX, DOXIL, or ThermoDox) at various doses (i.e., 2.5, 5, or 10 mg/kg) or saline. Only mice receiving ThermoDox formulation were treated with hyperthermia for 1 hour immediately after ThermoDox administration. Subsequently, mice were either used to study the efficacy (*N* = 5 or 6 per formulation per dose) or to study the drug distribution 24 hours after treatment (*N* = 3 per formulation) (Figure S5).

### 4.5. Hyperthermia Treatment

For hyperthermia treatment, mice were anesthetized with isofluorane (1.5–2%) immediately after ThermoDox administration. Subsequently, the tumor and the tumor bearing leg were covered with Vaseline and submerged in a water bath (WNE14l, Memmert, Schwabach, Germany) of 42 °C for 1 hour (Figure S2A).

In a separate experiment, intra tumor and core (rectal) temperatures were monitored by calibrated fiber optic temperature probes (Neoptix Reflex, Neoptix, QC, Canada, LP). Within one minute, tumors reached a temperature of 42.0 ± 0.19 °C, which was similar to the water bath temperature and remained constant over time. The core temperature was 36.9 ± 0.18 °C during the treatment (Figure S6).

### 4.6. Efficacy Study

Mice were weighed and tumors were measured three times a week. Tumor volume was determined using the following equation: 0.5 × length × width × width. Mice were monitored until 5 times the initial tumor volume (tumor volume during treatment) was reached or until 60 days after treatment.

### 4.7. Drug Distribution Study

Mice were intraperitoneally (i.p.) injected 60 and 20 minutes before euthanasia with 0.5 ml pimonidazole (hypoxia marker, 4 mg/mL in saline, Hypoxyprobe, Burlington, MA, USA) and 0.5 mL BrdU (s-phase marker, 3 mg/mL in saline, Sigma), respectively. One minute before euthanasia, mice were i.v. injected with 0.1 mL Hoechst 33342 (perfusion maker, 3.25 mg/mL in saline, Sigma). Subsequently, euthanasia was performed by cervical dislocation. Tumors were harvested, embedded in optimal cutting temperature (OCT) compound, snap frozen in liquid nitrogen, and stored at −80 °C.

### 4.8. Immunohistochemistry

Tumors were cut in 5 µm thick slices, mounted on glass, air-dried, and stored at −80 °C until further use. Before immunohistochemistry staining, the fluorescence of doxorubicin and Hoechst was recorded, as described below. Subsequently, two successive slices were stained for CD31 and pimonidazole and for BrdU [67]. First, all slices were fixed in acetone at 4 °C for 10 minutes and rehydrated in PBS. Staining of CD31 and pimonidazole was performed by incubating slices overnight with rat anti-mouse CD31 (1:50, 550274 BD pharmingen, San Diego, CA, USA) and rabbit anti-pimonidazole (1:500, hypoxyprobe) at 4 °C in a humidified chamber. Then, they were incubated for 1 hour with goat anti-rat Cy5 (1:500, ab6565, Abcam, Cambridge, UK) and goat-anti-rabbit IgG H&L Alexa fluor®594 (1:500, ab150080, Abcam) at room temperature in a humidified chamber and finally mounted with Fluoromount W (2163401, Serva electrophoresis GmbH, Heidelberg, Germany). Staining of BrdU was performed by incubating slices for 10 minutes in 2N HCl and neutralized for 10 minutes in 0.1 M Borax for antigen retrieval. Subsequently, slices were incubated overnight with sheep anti-BrdU (10 mg/ml, GTX21893, GeneTex, San Antonio, TX, USA) at 4 °C in a humidified chamber. Then, they were incubated for 1 hour with donkey anti-sheep IgG H&L Alexa fluor®594 (1:200, ab150180, Abcam) at room temperature in a humidified chamber, incubated with Hoechst 33342 (1 µg/mL) for 1 minute, and mounted with Fluoromount W. Between all incubations, the slices were washed 3 times for 5 minutes in PBS. All antibodies were diluted in primary antibody diluent (PAD, GTX28208 GeneTex).

### 4.9. Image Acquisition and Processing

Whole tumor slices were scanned by Leica TCS SP8 X microscope (Leica, Germany) at 10 times magnification. Before staining, the fluorescence (excitation, emission) was recorded for doxorubicin (504, 540–680 nm) and Hoechst (405, 410–499 nm). After staining, the fluorescence was recorded for CD31 (649, 665–750 nm), pimonidazole (598, 615–720 nm), and BrdU (598, 615–720 nm). For each slice and fluorophore, a z-stack was acquired, and a maximum intensity projection was created for further analysis. Images before and after staining were semi-automatic aligned, tumors were manually segmented, and artifacts and necrotic areas were manually removed. All processing and analyzing was performed in MATLAB (MathWorks, Inc, Natick, MA, USA). Example of total tumor and zoomed in detail of the stainings of the tumor treated with 5 mg/kg ThermoDox is presented in Figure S8.

Doxorubicin images were normalized for intensity fluctuations of the laser by dividing the tumor area by areas without any tissue. Since tumor autofluorescence can vary between tumors [68], autofluorescence of the tumor tissue was removed by subtracting the mean of the 10% smallest intensities of the normalized image.

Masks of vessels and dividing cells were obtained by applying a local maxima filter on CD31 and BrdU images, respectively. Masks of perfusion and hypoxia were acquired by interactive thresholding of the Hoechst and the pimonidazole images, respectively. The mask of perfused vessels was created from the overlay of the vessels and the perfusion mask [69]. A vessel was considered as perfused when both vessel mask and perfusion mask were positive for this vessel.

### 4.10. Image Analysis

Dividing cell fraction, vessel density, perfused vessel density, and hypoxic fraction were calculated on whole tumor slices by dividing the amount of positive pixels in each mask by the amount of pixels of the segmented tumor.

The doxorubicin heterogeneity parameter (Hdox) was determined over the whole tumor of the doxorubicin images to investigate the spatial doxorubicin distribution. First, tumor slices were binned by 10 × 10 pixels to reduce the matrix size of the image and cropped to avoid rim artifacts. Subsequently, the cropped image was filtered with a local mean filter of 51 × 51 pixels (0.7 × 0.7 mm). Finally, the standard deviation of the pixels in the filtered image was divided by the standard deviation of the unfiltered image (Figure S7A). Low values represented homogeneous spatial doxorubicin distribution, whereas high values represented heterogeneous spatial doxorubicin distribution.

The 90th percentile (P90) was used as a measure of the doxorubicin concentration in the whole tumor, i.e., 90% of all intensities of the doxorubicin image in the tumor were below this value. 

The mean fluorescent intensity of the doxorubicin signal (doxorubicin intensity) in relation to the distance of the perfused vessels was determined [54]. Briefly, the perfused vessel mask was converted into a distance mask. Subsequently, the distance mask and the doxorubicin image were superimposed, and the mean doxorubicin intensity was calculated for each distance (Figure S7B).

Finally, spatial correlation between the doxorubicin intensity and the dividing cells was determined by dividing each tumor image in squares of 200 × 200 pixels. For each square, the mean doxorubicin intensity and the dividing cell fraction were determined. Subsequently, each square represented one dot in the scatter plot. Dividing cell fraction below 0.05 and doxorubicin intensity below 0.5 were considered low. This resulted in 4 quadrants in the scatter plot, i.e., low/high dividing cell fraction in combination with low/high doxorubicin intensity.

### 4.11. Statistical Analysis

All tumor growth curves were presented as mean, with error bars representing the standard error of the mean. Kaplan–Meier curves were statistically tested in GraphPad Prism 7 (Graph-Pad Software, Inc, San Diego, CA, USA) by comparison of groups with a log rank Mantel–Cox test. Differences between groups with *p* < 0.05 were considered statistically significant.

## 5. Conclusions

At low and intermediate doses, the largest growth delay was obtained after ThermoDox treatment combined with hyperthermia, whereas at the largest investigated dose, the largest growth delay was obtained after DOXIL treatment. At each investigated dose, tumors treated with ThermoDox contained higher doxorubicin concentrations compared to DOXIL and DOX. For all formulations, at the largest dose, the spatial doxorubicin distribution was most homogeneous and the doxorubicin concentration in the tumor was the largest. On histology, tumor areas with high doxorubicin concentrations corresponded with areas with low cell proliferation. Unfortunately, no distinct relation between tumor growth and drug distribution and concentration was found. This is most likely related to changes in spatial drug distribution over time, which were not investigated in this study. 

## Supplementary Materials

The following are available online at https://www.mdpi.com/2072-6694/11/10/1512/s1, Figure S1: Different stainings of tumors treated with DOX of 2.5, 5 and 10 mg/kg, Figure S2: Different stainings of tumors treated with DOXIL of 2.5, 5 and 10 mg/kg, Figure S3: Different stainings of tumors treated with ThermoDox of 2.5, 5 and 10 mg/kg, Figure S4: Doxorubicin intensity relative to the perfused vessels in locations with high and low doxorubicin intensities, Figure S5: Experimental timeline of the experiments, Figure S6: Hyperthermia setup, Figure S7: Calculations of Hdox over the whole tumor and doxorubicin intensity in relation to the perfused vessels, Figure S8: Total tumor and zoomed in detail of the stainings of the tumor treated with 5 mg/kg ThermoDox; doxorubicin (A), Perfusion (B), All vessels (C), hypoxia (D) and dividing cells (E). The scale bar in the total tumor represents 1 mm. The size of the zoom is 1.43 × 1.43 mm.
