# Supplementary material for: Tumor Drug Distribution after Local Drug Delivery by Hyperthermia, In Vivo
Source: Cancers (Basel). 2019 Oct 9;11(10):1512. doi: 10.3390/cancers11101512 (PMC6826934; doi:10.3390/cancers11101512)
Supplement: Supplementary file 1 [file cancers-11-01512-s001.pdf]

Supplementary Materials:

Tumor Drug Distribution after Local Drug Delivery by Hyperthermia, In Vivo

Helena C. Besse, Angelique D. Barten-van Rijbroek, Kim M.G. van der Wurff-Jacobs, Clemens Bos, Chrit T.W. Moonen and Roel Deckers

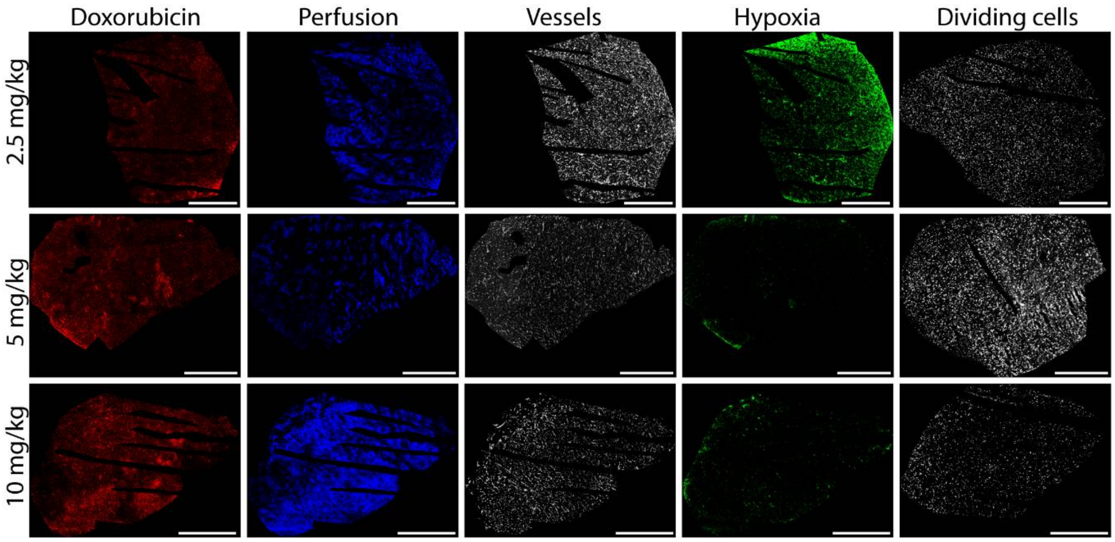

Figure S1. Different stainings of tumors treated with DOX of 2.5, 5 and 10 mg/kg. Tumors stained for doxorubicin, perfusion, all vessels, hypoxia and proliferating cells. The scale bar is 1 mm.

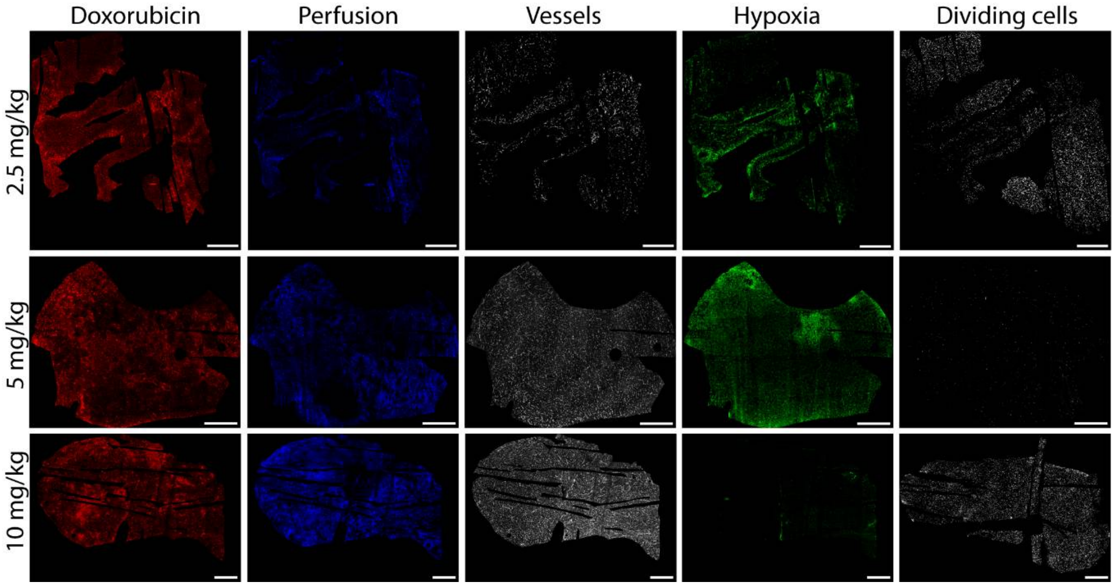

Figure S2. Different stainings of tumors treated with DOXIL of 2.5, 5 and 10 mg/kg. Tumors stained for doxorubicin, perfusion, all vessels, hypoxia and proliferating cells. The scale bar is 1 mm.

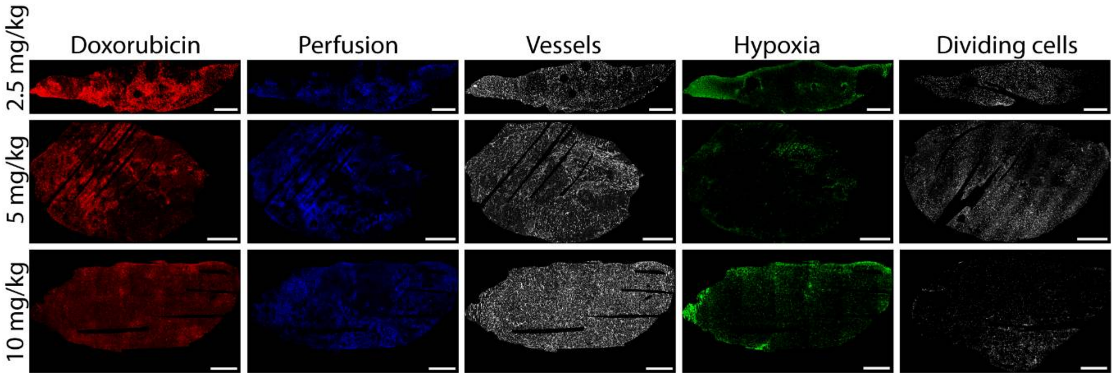

Figure S3. Different stainings of tumors treated with ThermoDox of 2.5, 5 and 10 mg/kg. Tumors stained for doxorubicin, perfusion, all vessels, hypoxia and proliferating cells. The scale bar is 1 mm.

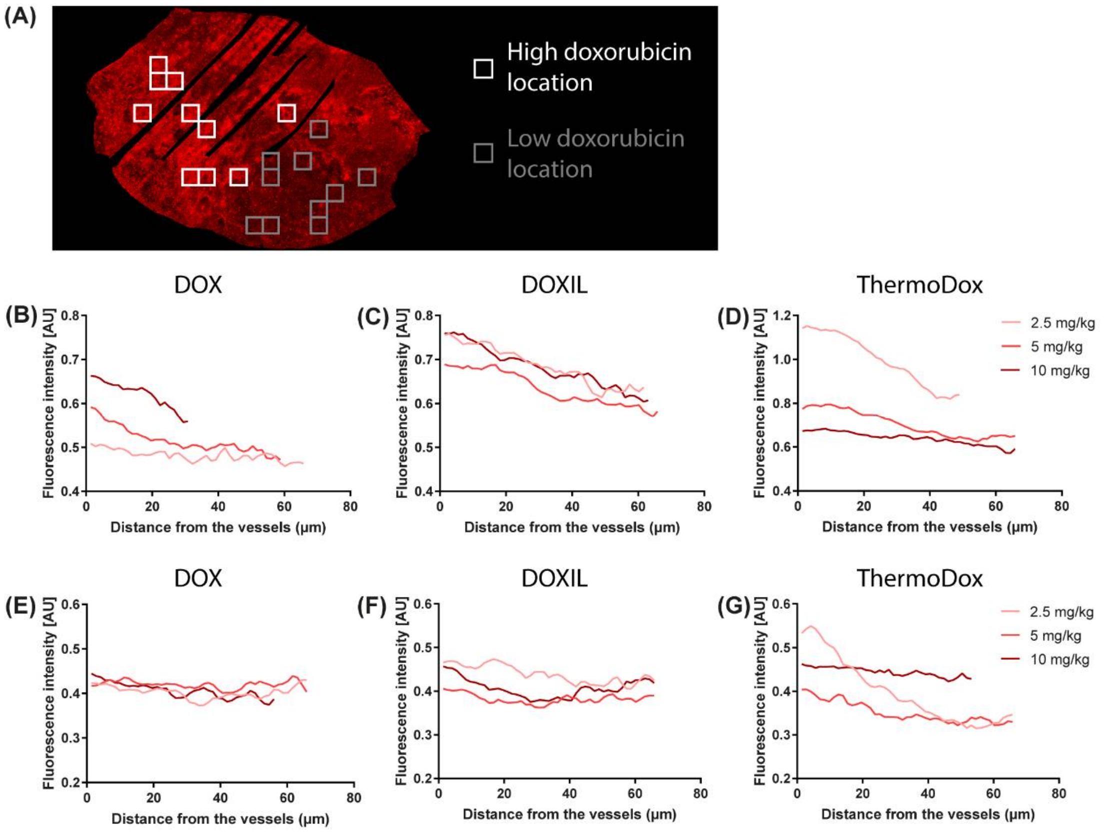

Figure S4. Doxorubicin intensity relative to the perfused vessels in locations with high and low doxorubicin intensities. Locations with high and low doxorubicin intensities were selected in each tumor (**A**). Doxorubicin intensities relative to the vessels in areas with high doxorubicin intensities after DOX (**B**), DOXIL (**C**) and ThermoDox (**D**) treatment and doxorubicin intensities relative to the vessels in areas with low doxorubicin intensities after DOX (**E**), DOXIL (**F**) and ThermoDox (**G**) treatment.

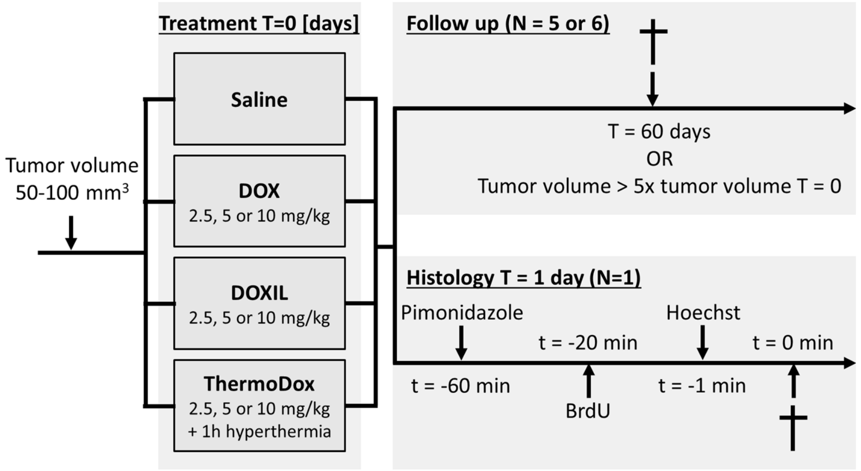

Figure S5. Experimental timeline of the experiments. Mice with tumor volumes between 50 and 100 mm3 were treated with saline, DOX, DOXIL or ThermoDox at a dose of 2.5, 5 or 10 mg/kg. Only after ThermoDox administration tumors were treated for 1 hour with hyperthermia. Subsequently, mice were used to study the efficacy or to study the doxorubicin distribution 24 hours after treatment.

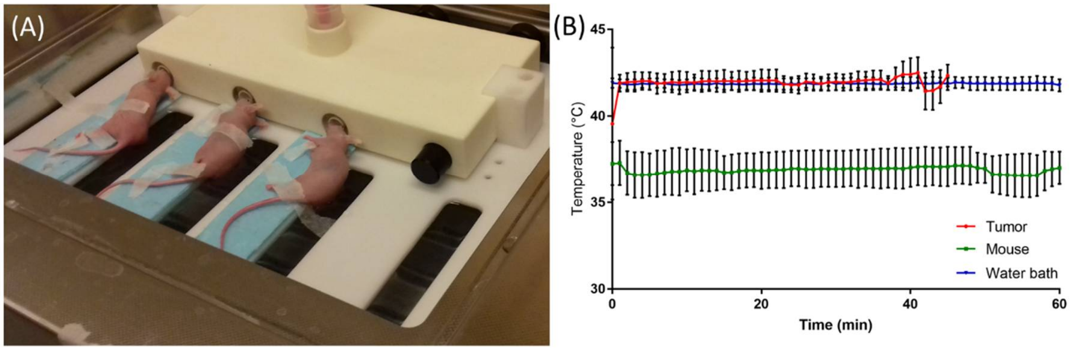

Figure S6. Hyperthermia setup. A photo of the hyperthermia setup (**A**) and temperature measurements of tumor (*N* = 4), mouse (core) (*N* = 5) and water bath (*N* = 5) during hyperthermia treatment up to 60 minutes (**B**)

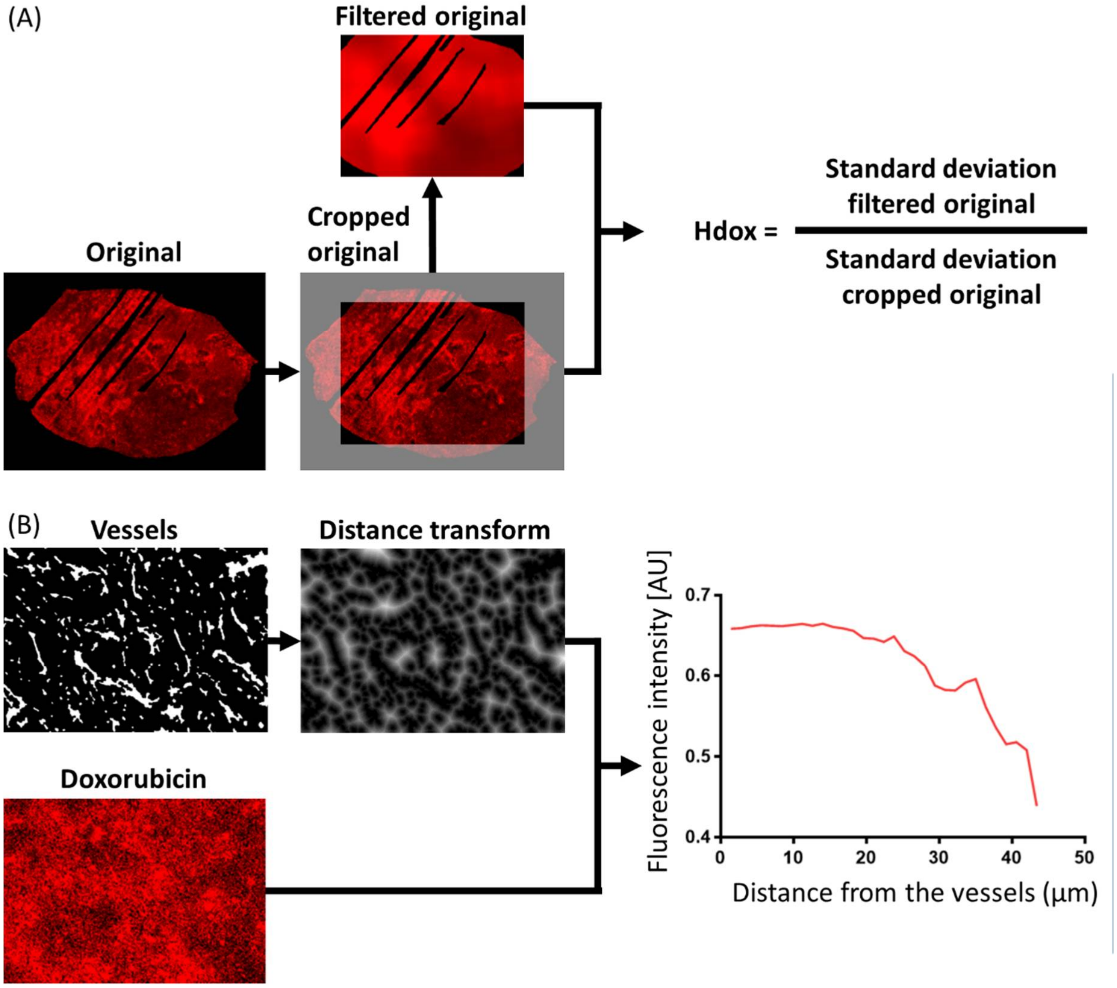

Figure S7. Calculation of Hdox over the whole tumor and doxorubicin intensity in relation to the perfused vessels. Hdox (**A**) was calculated by cropping the original image, subsequently the cropped image was filtered and the ratio of the standard deviation of the filtered image and cropped image was calculated. The doxorubicin intensity in relation to the vessels (**B**) was calculated by converting the perfused vessel mask to a distance map, and then combining the distance map and doxorubicin image to obtain the average fluorescence intensity as a function of the distance from the perfused vessels.

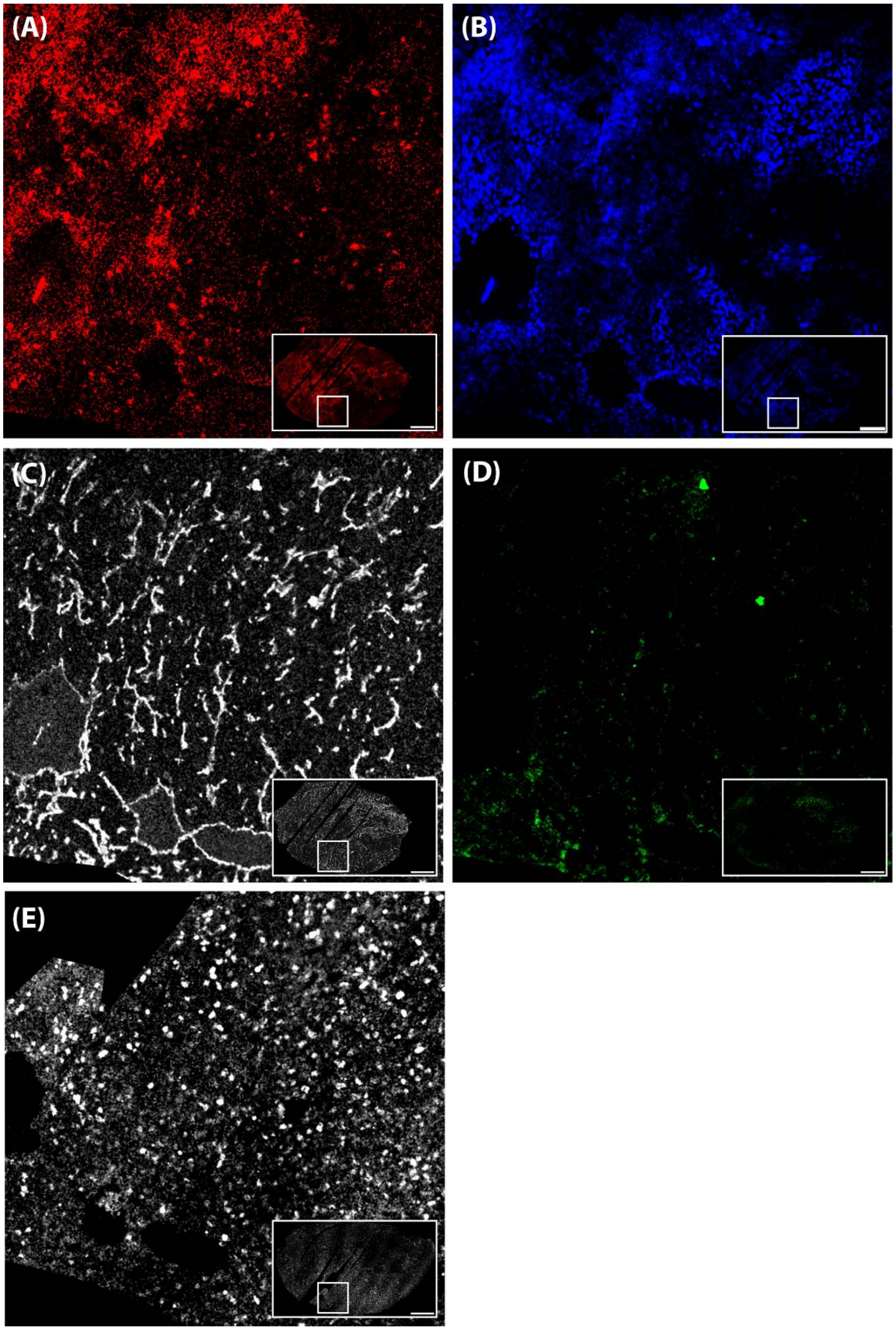

Figure S8: Total tumor and zoomed in detail of the stainings of the tumor treated with 5 mg/kg ThermoDox. doxorubicin (**A**), Perfusion (**B**), All vessels (**C**), hypoxia (**D**) and dividing cells (**E**). The scale bar in the total tumor represents 1 mm. The size of the zoom is 1.43 × 1.43 mm.

© 2019 by the authors. Licensee MDPI, Basel, Switzerland. This article is an open access article distributed under the terms and conditions of the Creative Commons Attribution (CC BY) license (http://creativecommons.org/licenses/by/4.0/).
